# Virtual and Artificial Cardiorespiratory Patients in Medicine and Biomedical Engineering

**DOI:** 10.3390/membranes12060548

**Published:** 2022-05-25

**Authors:** Krzysztof Zieliński, Tomasz Gólczewski, Maciej Kozarski, Marek Darowski

**Affiliations:** Nalecz Institute of Biocybernetics and Biomedical Engineering, Polish Academy of Sciences, 02-109 Warsaw, Poland; tgolczewski@ibib.waw.pl (T.G.); mkozarski@ibib.waw.pl (M.K.); mdarowski@ibib.waw.pl (M.D.)

**Keywords:** modeling and simulation, cardiopulmonary interaction, gas exchange, hybrid model, artificial patient, virtual patient, membrane-based cardiovascular support systems, extracorporeal membrane oxygenation

## Abstract

Recently, ‘medicine in silico’ has been strongly encouraged due to ethical and legal limitations related to animal experiments and investigations conducted on patients. Computer models, particularly the very complex ones (virtual patients—VP), can be used in medical education and biomedical research as well as in clinical applications. Simpler patient-specific models may aid medical procedures. However, computer models are unfit for medical devices testing. Hybrid (i.e., numerical–physical) models do not have this disadvantage. In this review, the chosen approach to the cardiovascular system and/or respiratory system modeling was discussed with particular emphasis given to the hybrid cardiopulmonary simulator (the artificial patient), that was elaborated by the authors. The VP is useful in the education of forced spirometry, investigations of cardiopulmonary interactions (including gas exchange) and its influence on pulmonary resistance during artificial ventilation, and explanation of phenomena observed during thoracentesis. The artificial patient is useful, inter alia, in staff training and education, investigations of cardiorespiratory support and the testing of several medical devices, such as ventricular assist devices and a membrane-based artificial heart.

## 1. Introduction

Several governmental agencies and non-profit organizations, such as the Avicenna Alliance, the Centre for Devices and Radiological Health of the U.S. Food and Drug Administration, and the European Union (Directive 2010/63/EU), for example, have encouraged ‘in silico trials’ to reduce animal experiments. In particular, there was an idea to create the Virtual Physiological Human [1]. Additionally, in silico experiments can decrease the cost of the research and development of medical devices [2]. They play also an important role in the process of developing new drugs [3] and allowing an understanding of various phenomena at the molecular level [4]. Computer models can be used in medical education, biomedical research (for example, to study particular cardiorespiratory phenomena) and in clinical applications (for example, to aid medical procedures and optimize the patient’s treatment) [5]. However, although computer models may be useful in medicine and biomedical engineering, they have one great disadvantage: no possibility to interact with physical devices, e.g., with devices for organ functions support. Therefore, for ‘in silico’ investigation of relations between a particular device and an organ, a numerical model of this device would be necessary. Such a model, unfortunately, can be only an approximation of the real device, particularly of a new tested device, unless instead of the device, only a method of support is investigated. Physical models do not have this particular disadvantage; however, they have a number of other ones, e.g., they are uneasily modifiable and expensive, and they usually cannot mimic biological systems with the desired precision, or in most cases, cannot mimic some biological phenomena, such as O_2_ and CO_2_ transfer across the respiratory tract, their exchange by gas-permeable membranes in alveoli and tissues, and transport through the cardiovascular system. These phenomena can be easily implemented in numerical models.

To avoid the above problems, Pillon et al. presented the idea of connection of numerical circulatory models and a real ventricular assist device (VAD) by means of a unique interface [6]. Verbraak et al. proposed a bellows-based respiratory system simulator with a computer-controlled system mimicking the diaphragm [7]. These works led to the term ‘hybrid model’ or ‘hybrid simulator’ since such models or simulators consist of two different components: numerical and physical [8]. Certainly, there has to be a third component, i.e., a physical–computer interface, which is the crucial element in hybrid modeling.

There are a number of aspects of a biological system which are difficult to be modeled physically, such as the neural control, pharmacological agents or mentioned above membrane-based phenomena, such as gas exchange, for example. Therefore, the system element that interacts with a physical device has to be modeled physically, whereas the others can be modeled either physically or numerically, and the constructor of a hybrid simulator should determine which method should be chosen for a particular element or aspect. Such features as costs, required model flexibility as well as reliability and the accuracy of mathematical description necessary in investigation, for example, should be taken into account. In general, it is strongly recommended to minimize the number of parts modeled physically in order to maximize benefits of computer modeling. A numerical component may be either relatively simple or complex, which depends on an analyzed problem. The use of a very complex general purpose model of a biological system (‘virtual patient’) is another choice. A hybrid model with such a virtual patient as its numerical component can be called an ‘artificial patient’.

The chosen approach to the cardiovascular system (CVS) and/or respiratory system (RS) modeling are discussed in this review, with particular emphasis given to the artificial patient that was elaborated by the authors.

## 2. Approaches to Cardiovascular and Respiratory Systems Modelling

Due to the increasing role of modeling in medicine and biomedical sciences, there have been developed thousands of models of the CVS and RS. Because of this great number of various models, only the types of approach to modeling can be discussed here. Depending on the used criteria, the models can be differently grouped.

First of all, a group of 3D models reflecting anatomical geometry of *CVS* or *RS* can be identified. Recently, physical 3D models, usually 3D-printed models, are the most frequently used to aid surgical and diagnostic procedures (e.g., [9,10,11]) or in medical education and staff training (e.g., [12]); however, some of them are used for research of a chosen phenomenon (e.g., [13]). Here, it should be mentioned a very interesting 3D model of lungs containing living cells, which were infected with real respiratory syncytial viruses [14]. There are also a number of 3D or 2D computer models, e.g., an interesting connection of 2D model of the heart with a simple 0D model of the vascular system [15].

Although 3D models constitute a group that is very useful in medicine and education, only models simulating the RS and/or CVS work are discussed below. Note, however, that there are models, which, though they simulate the work, some geometrical relations are taken into account to make it possible to simulate the ventilation–perfusion mishmash, for example, caused by gravity, pleural effusion, etc. (e.g., [16,17]). Additionally, although a CVS and/or RS model belongs to 0D models, in general, more precise simulations of gas transfer and transport with blood require 1D sub-models to simulate the propagation of changes in gas tensions with realistic, finite velocity (e.g., changes caused by altered minute ventilation [18]). For that reason, bronchi and vessels in such models require description by discrete differential equations. Additionally, simulations of elements, being both significantly resistive and significantly compliant, e.g., pulmonary capillaries or medium-sized bronchi, require a kind of 1D representations [19].

Taking into account applications, several groups of models simulating the CVS and/or RS work can be distinguished, e.g., those used in education, research or medical procedures support. The existence of educational models that are based on pure medical knowledge can be only mentioned here despite their complexity (e.g., [20,21]) because they are usually not based on a mathematical description of physical properties of the RS and/or CVS. In general, since these properties are nonlinear, they should be mathematically described by nonlinear equations rather than by simple numbers, such as ‘airway resistance’ or ‘pulmonary vascular resistance’ (additionally, the values of those resistances at a given moment depend on several factors and variables, such as pleural pressure, for example). If, however, a model is intended to be used in simulations of responses to small stimuli, simple numbers can be used. For example, forced spirometry or explanations of phenomena observed during thoracentesis [17,19] require nonlinear equations (note that airflow does not depend on the driving pressure during forced expiration and thus the Ohm’s idea of resistance loses meaning); however, simulations of tidal breathing may use simple numbers, e.g., those proposed by Arnal et al. [22].

From the technical point of view, all models can be used in education if they have a user-friendly interface (e.g., [16,23,24]). On the other hand, some simulation-based environment originally developed for learning about physiology, can be utilized in research, e.g., the educational Harvi environment [25] was utilized in a model used to investigate the optimal form of circulatory support in a ventricular septal defect [26].

A number of the CVS and RS computer models have been developed to solve only a particular problem (e.g., [27]) or to support a medical procedure in cases of individual patients. The latter models, called patient-specific models or bedside models, are of special meaning and have been used for dozens of years. Indeed, vascular or airway resistance and arterial or lung compliance, for example, are the simplest models (the RC circuits) used in medicine, in fact, despite that physicians usually do not recognize that they use simple mathematical models. Parameters of those models and such original variables as the cardiac output and arterial pressures constitute the fundamental set of quantities characterizing the patient’s state. Certainly, development of the IT technology enabled to develop more complex models, giving much more information and predictions [28,29,30,31]. Identifiability from the data available at the bedside or clinical situation is a main feature of such models; however, this is also the main limitation of those models: they cannot be too complex. For example, local pleural pressures, which have significant influence on both ventilation and the perfusion of particular lung regions, cannot be measured and, in consequence, models containing these pressures rather cannot be used as patient-specific models. Note that there are patient-specific models of the RS (e.g., [32]) but their number is far lower that the number of patient-specific models of the CVS.

Research with the use of models may concern either the average human being or a virtual/artificial population. This population can be created in two main ways, in general [33]. The first method consists of deviation of the model parameters from their values assumed for the average human being (e.g., [34]). A 1-to-1 mapping approach, in which each virtual patient corresponds to a real patient is the second method, i.e., the virtual population consists of patient-derived virtual patients. Certainly, the second approach is possible if a model is a simpler, patient-specific model rather than a more complex one.

More detailed comparison between particular CVS or RS models would be impossible due to their number. However, as an example, the following seemingly very similar works can be compared: the tests presented by Kung et al. [35] and the investigation presented by Di Molfetta et al. [36]. Both works concerned Jarvik VADs, and own hybrid models were used in those works. Thus, both the methodology and the kind of medical devices were the same. Certainly, there were also significant differences. First of all, these works concerned different VADs: the Infant Jarvik 2015 was studied by Di Molfetta et al., whereas Kung et al. used the Jarvik 2000 for adults. Moreover, Di Molfetta et al. tested the Infant Jarvik 2015 with the use of their own hybrid model, whereas Kung et al. tested their own hybrid model with the use of the Jarvik 2000.

The latter difference was caused by the fact that Kung et al. proposed a new approach to coupling the numerical and physical components, and therefore they wanted to test it. The original, hardware-in-the-loop approach proposed by Pillon et al. [6] and Verbraak et al. [7] has been commonly used by several authors (e.g., [37,38,39,40]). According to this approach, a change in the physical component creates the corresponding reaction of the numerical component and vice versa. Therefore, numerical simulation of physiological phenomena during a time period, usually equal to 1 ms, has to take less than this period to make it possible to synchronize the real-time and simulated ones. Therefore, the hardware-in-the-loop approach requires real-time systems for numerical simulation, and computer models requiring time-consuming numerical calculations cannot be numerical components of hybrid models because the above synchronization would be impossible. Kung et al. [35] proposed another approach to overcome those limitations. According to this approach, an iterative coupling method to achieve the dynamic closed-loop feedback between the physical and numerical domains is used. This iterative algorithm modulates the common flow waveform(s) to identify their shapes, when pressure drops in the physical part (called a physical experiment) are equal to respective pressure drops in the numerical part (called computational physiology simulation). The common flow waveforms mean the flows connecting two domains and existing both in numerical and physical forms.

This new approach was further developed to enable more complex experiments [41]. As the absence of real-time calculations is the main advantage of this approach, hardware bandwidth limitations have minimal restraint. Thus, the time-consuming operations can be used in hybrid models built in accordance with Kung’s concept, whereas time-consuming 3D calculations, for example, are impossible in the case of hybrid models built in accordance with Pillon’s concept (pending a significant increase of the computers speed). Unfortunately, the Kung’s concept has one great disadvantage: it requires a kind of steady-state condition of simulations, as each change of the conditions requires new initial iterations. For that reason, this approach is not useful in simulations of changes, e.g., adaptive mechanisms in mechanical circulatory or ventilatory support devices or the left ventricular suction and the rotary LVAD speed reduction to prevent this phenomenon.

An extracorporeal membrane oxygenation (ECMO) system is reserved as a last resort of life support technology and is used when treatment by a mechanical ventilator and other strategies fail. In cases of respiratory failure ECMO may be applied for few days only while lung disaster caused by, for example, COVID-19, may require at least 4 or many more weeks to effectively help the sickest patients. ECMO is the ultimate tool to replace lungs or/and heart functions when one of these organs do not work anymore. The task of ECMO is to transfer the gas exchange from these organs to an extracorporeal membrane for oxygenation and CO_2_ removal. It gives time to heal the patient’s organ or to replace it by an implant. ECMO is supportive therapy, not a disease-curing treatment. ECMO application is a complex and very costly process that requires well-trained professionals and, being the most invasive treatment, at the same time lowers risk of death due to, for example, COVID-19 in critically ill patients—both adults and children. ECMO was also successfully used at the outbreak of an influenza *A* (*H1N1*) in 2013. ECMO is a medical technology that has two applications: for cardiovascular support (by venous–arterial (*V-A*) cannulation) and for treatment of acute respiratory failure (by veno–venous (*V-V*) cannulation). An enormous increase of ECMO centers in recent years was observed [42] and in order to increase patients’ safety and to optimize ECMO efficacy, both training simulators as well as hybrid simulators are needed [43,44,45,46,47,48]. The simulators can help in ECMO system training, enabling, in some cases, high fidelity clinical scenarios [43,44] or to study the hemodynamic effect of ECMO on the left ventricular loading in a *V-A* configuration and the parasitic effect of blood recirculation in *V-V* cannulation [46]. However, these simulators usually can only mimic the hemodynamic physical effect when connected to an actual ECMO. The membrane oxygenation and CO_2_ removal usually must be simulated numerically due to the fact that oxygen consumption and carbon dioxide production in cardiopulmonary physiology are difficult to simulate physically. One of exceptions is the simulator presented in [47] using the hardware-in-the-loop approach with a physiological numerical model, where a computer-controlled de-oxygenator was utilized to simulate the blood flow with a desired oxygen level entering the ECMO system.

One of the major limitations of artificial as well as virtual patients (models) is their validation. It is important especially when they are considered as a part of decision support systems when simulating the specific patient. The validation procedure of the model usually is as follows [5]. The patient’s data, such as characteristic and diagnostic data and therapeutic actions are inputs for the model. The model simulates specific patients then, and its output data are compared with the outputs of real patients. However, it requires a large-scale database [5] with data from various sources of intensive-care units. An artificial intelligence could support this validation process (for example, adjusting the model’s parameters), as it can be applied in clinical examinations and diagnosis [49]. If the model is not used to simulate the specific patient but rather to study phenomena or for training and education purposes, a simpler verification can be enough. An exemplary process of cardiovascular model verification in case of the left ventricular assistance in animals was presented in [50].

The CVS and RS have a common goal (oxygen delivery to tissues), and their failures have frequently common symptoms (e.g., dyspnea). To accurately test physical devices for ventilatory or cardiac support, an artificial patient has to contain an accurate model of the RS with pulmonary circulation, which can simulate such phenomena that are important for blood saturations as ventilation–perfusion mismatch or influence of obstructive lung diseases of any severity on the CVS work, for example. The artificial patient applied by the authors has this potential [51], which led to the creation of the CARDIOSIM© simulator [28,29,48], which can be used in medical devices testing [8]. As the numerical components of this artificial patient are 0D models, in general, they cannot be used in investigations of phenomena at the microscale, such as turbulences in air and blood flows, for example. However, they are good and partly exceptional tools to investigate a wide range of phenomena at the macroscale. In particular, although physical equipment testing was the original goal of the artificial patient, the models were utilized in systems for e-learning and e-support of medical decisions and used in analyses of cardiopulmonary interaction in different cases (see Appendix A for examples). As, to the authors’ knowledge, this artificial patient is the most general-purpose platform for various simulations, it is described below in more detail.

## 3. Cardiovascular and Respiratory Models Elaborated by the Authors

### 3.1. Preliminary

**Figure 1:**
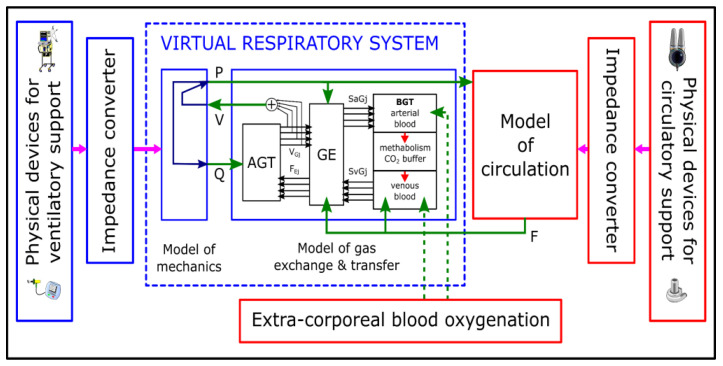
The idea of the cardiopulmonary hybrid platform developed by the authors. As oxygen delivery and carbon dioxide removal are the fundamental goals of the cardiorespiratory system, the model of gas transfer and exchange is the central point (it consists of modules of AGT—airways gas transfer, GE—gas exchange, BGT—blood gas transport). Respiratory system mechanics influences AGT, GE, and circulation. Cardiovascular system mechanics influences BGT and GE. Respiration and circulation support as well as oxygenation/decarbonation can be simulated or realized with physical devices by means of impedance converters playing the role of numerical–physical interfaces. V, Q, P and F denote volumes, airflows and pressures in the respiratory system, and blood flows, respectively.

The cardiopulmonary hybrid platform (Figure 1), which has been developed by the authors for years [52], is composed of a hybrid respiratory simulator (HRS) and a hybrid cardiovascular simulator (HCVS). They can work separately or can be interconnected by the collaboration of their numerical components. Depending on purposes, either simple or very complex numerical models can be used as the numerical parts of the simulator. For example, if the interaction between the RS and CVS is not considered when the HCVS is used, then RS need not be simulated, and a model of pulmonary circulation can be very simple, e.g., composed of one resistive compartment and one compliant compartment simulating the total resistance and compliance of pulmonary vessels, respectively. On the other hand, if only the RS work is investigated, e.g., the ventilation of preterm infants [53] or forced spirometry (see the Appendix A), then either no CVS model is required, or it can be simple. If, however, blood gases are investigated, for example, then both RS and CVS models are required.

Since gas tensions in blood depend on gas exchange, and gas exchange depends on lung ventilation, pulmonary circulation and the ventilation/perfusion ratio in particular lung regions, the pulmonary circulation has to be simulated by a RS model (Figure 1). Therefore, the HRS of the most complex version contains a virtual patient, i.e., a set of models of the RS mechanics, pulmonary circulation, gas transfer in bronchi, gas exchange in lungs and gas transport with blood in both pulmonary and systemic circulations (Figure 1). The models of pulmonary circulation and gas transport in the systemic circulation use data related to blood flows in systemic vessels and pressures in the right ventricle and left atrium, delivered by a CVS model. On the other hand, the HCVS may use necessary data from the HRS unless a simple embedded model of pulmonary circulation is used. Depending on a particular application of the HCVS, chosen parts of a CVS, e.g., a part of the aorta, can be simulated by physical elements. In the case of the HRS, only the input of the bronchial system was realized physically until now.

### 3.2. Numerical Components

#### 3.2.1. Virtual Patient

A set of models of the RS mechanics, gas transport end exchange, and pulmonary circulation was developed and used in several applications (see the Appendix A for examples). Their main features are briefly described below (details are presented elsewhere (e.g., [16,17,18,19,54]).

The lungs, chest wall and mediastinum are modeled separately. In the last applications, the chest wall was decomposed into the rib cage, two hemidiaphragms and the abdomen [54]. Lungs are divided into several parts, e.g., into 80 parts in the basal version, which concerns both the RS mechanics and pulmonary circulation models. This enables to simulate ventilation–perfusion mismatch [16] or investigate pulmonary shunt during thoracentesis (see Appendix A), for example. The hypoxic pulmonary vasoconstriction was introduced in [17]. It joins local oxygen tension with the lumen of the distal arteries to redistribute the blood flow from poorly oxygenated regions into well-ventilated ones.

Almost all elements are described by nonlinear equations; some of them are dependent on actual values of respiratory or circulatory variables. In particular, pulmonary capillaries and bronchi, being significantly resistive and compliant, are modeled, from a purely mathematical point of view, as distributed parameter elements. However, a suitable mathematical description of these elements enables to solve differential equations manually (analytically) [19], and the solutions are used in computer models as seemingly lumped parameter elements. For example, the lung compliance (Equation (1)) with physiologically interpretable parameters and the resistance of collapsible bronchi (Equation (2)) are described by the following equations:

(1) $$\mathrm{Ptps}=\tan\left(\left(\pi /2\right)\cdot \left(V_{A}-V_{A0}\right)/\mathrm{Size}\right)/\mathrm{Compl}$$

(2) $$f=\frac{\mathrm{Pb}-\mathrm{Pa}-b\cdot \mathrm{arctg}\left(b\cdot \frac{\mathrm{Pb}-\mathrm{Pa}}{b^{2}+\left(\mathrm{Pb}-\mathrm{Pp}\right)\cdot \left(\mathrm{Pa}-\mathrm{Pp}\right)}\right)}{k}$$

where Ptps is the static transpulmonary (recoil) pressure; V_A_ is the lung volume; V_A0_ is the volume when the recoil pressure is equal to zero; Size is the parameter which may be treated as the size of lungs (for example: the Size value depends on patient’s height, and resection of a lung part results in a corresponding decrease of the Size value); Compl is the parameter that describes the compliant properties of the lung tissue (it can be treated as the tissue unitary compliance at V_A0_); f is the airflow through collapsible bronchi; Pp, Pa and Pb are the pleural, alveolar and bronchial pressures, respectively; the parameter b characterizes compliant properties of those bronchi; and k characterizes their resistive properties (note that the current ‘ohmic’ airway resistance (Pb − Pa)/f depends on several factors and is close to k only in cases of very weak expirations or inspirations at the total lung capacity). According to the spirometric reference values for the Polish population [55], parameters of the basal set of the equation parameters were adjusted to average middle-aged Polish women [16].

See the Supplementary Materials in [17] for nonlinear equations used in the pulmonary circulation model.

#### 3.2.2. Cardiovascular System Models

Two different heart models were used. The first one is based on the Frank–Starling law, and the time-varying elastance concept for systolic phase and an exponential pressure–volume relationship for ventricular filling [56,57]. The second heart model [58] additionally introduces the ECG signal; thus, pathophysiological conditions, such as bundle branch block, can be simulated. Moreover, it takes into account interventricular septum and active atria.

Additionally, two models were used to simulate the systemic circulation. The basic one consists of a one-element Windkessel for the arterial circulation and another one-element Windkessel for the venous circulation [57]. The venous return is implemented according to Guyton’s curve. A more advanced model is based on a multi-compartment approach, including several circuits: ascending aorta and aortic arch, descending aorta, upper body vessels, kidney, liver and splanchnic vessels, and lower limbs [59]. Each compartment consists of a one-element Windkessel. The second model may cooperate with a baroreflex control system model [50]. The switched-on mode affects ventricular contractility, heart rate and peripheral resistance.

### 3.3. Hardware and Physical Components

#### 3.3.1. Respiratory System

The newest double-piston version of the HRS (based on the solution presented in [60]—Chapter 8) gives about 5 liters of the total working volume. They are commonly driven by the direct current (DC) motor coupled with a ball screw. The DC motor is combined with the tachogenerator and is controlled by a servo amplifier. The electro-pneumatic actuator (pistons) is equipped with a linear displacement transducer to control the pistons’ positions. A pressure transducer is connected to the pistons’ chambers. All hardware is deployed in the compact chassis, including power supplies and a signals terminal for bidirectional communication with the computer system.

The HRS computer system consists of four core Intel processor-based industrial computer (PXI) with a data acquisition card installed with analog-to-digital and digital-to-analog converters on board. The PXI has a real-time operating system installed. It manages in real time the following tasks: data acquisition, control of the hardware, real-time simulation of the RS, and communication by transmission control protocol/internet protocol with a second computer (HOST). The HOST plays the role of a graphical user interface. The real-time computer PXI executes determinist computer operations, whereas the HOST executes nondeterministic operations such as visual ones, commends from the user, and data storing. This is a typical architecture in control-measurement systems to separate the time-critical functions from the others.

#### 3.3.2. Cardiovascular System

The HCVS hardware of the newest version consists of four separate chambers. Each of them is connected with a hydraulic gear pump driven by a direct current motor. Each chamber can be configured to reproduce one pressure in the cardiovascular system. Most of the mechanical circulatory support systems require two chambers (one for input and one for output) to connect them with the numerical model, e.g., the intra-aortic balloon pump requires two chambers and the small physical part that mimics descending aorta for insertion of the balloon. A total artificial heart device, however, requires all four chambers.

Just as in the case of the HRS, the numerical part of the HCVS was based on the PXI real-time system with the same data acquisition card installed. The HCVS also requires the HOST to communicate with users. See [59,61] for more details.

#### 3.3.3. Numerical—Physical Interface

**Figure 2:**
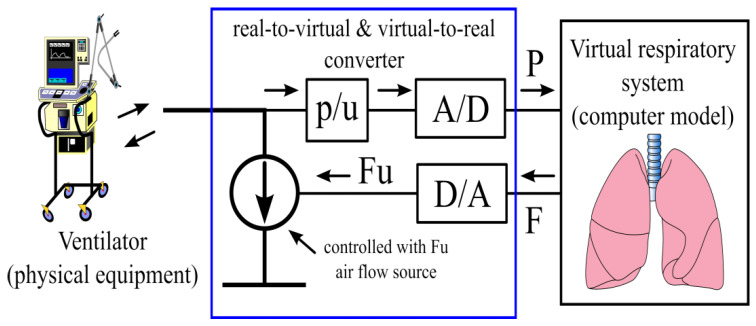
An example of the physical–numerical interface: A real ventilator ventilates the tube that simulates the trachea. Without respect to the type of a respirator and the support mode, the respirator work changes the pressure in the tube. That pressure is measured and converted to voltage ([p/U]), which after digitization ([A/D]) is the input (P) of a computer model. The model calculates the air flow (F) that would be for the measured pressure course in a real patient. This calculated value (converted to voltage Fu) controls the air flow source; in consequence, a quantity of the real air flows through the tube. Such flow causes a pressure change in the tube as though the air has gone to/from the real lungs. Thus, from the respirator point of view, it ventilates a real patient.

The interface is based on bidirectional signal transmission between the physical world (hydraulic or pneumatic in the case of the HCVS or the HRS, respectively) and the virtual one (computer models). It is equipped with an actuator to reproduce flows (or pressures) calculated by the computer model in the physical world, and to digitize pressures (or flows) being responses of physical parts of HCVS or HRS to the above flows (or pressures) [8,52]. Figure 2 presents an example of the interface.

### 3.4. Summary

As the models elaborated by the authors are 0D models, in general, they cannot be used in investigations of phenomena at the microscale, such as turbulences in air and blood flows. However, they are good and partly exceptional tools to investigate the phenomena at the macroscale. To the authors’ knowledge, for example, the presented HRS is the only hybrid model that enables to simulate the forced spirometry in the chronic obstructive pulmonary disease of all severities and etiology, thanks to the nonlinear mathematical description of the RS physiology, not as recorded airflow profiles in the form of previously measured curves. Certainly, simpler phenomena, e.g., related to spontaneous or supported tidal breathing or to cardiorespiratory interaction, can also be simulated by the same models. Appendix A presents the chosen applications.

## 4. Conclusions

There are many different models, and each of them is useful in some applications and unfit to be used for other purposes. Some models can be used in both research and education, the others are built to solve a particular problem. Relatively simple models are useful as patient-specific models, whereas complex models can be used in a wide range of problems without the necessity of their validation each time. In particular, a hybrid model with complex models as its numerical parts can be a good tool in biomedical engineering, as it enables to test a variety of new engineering achievements, e.g., new devices.

## Figures and Tables

**Figure A1:**
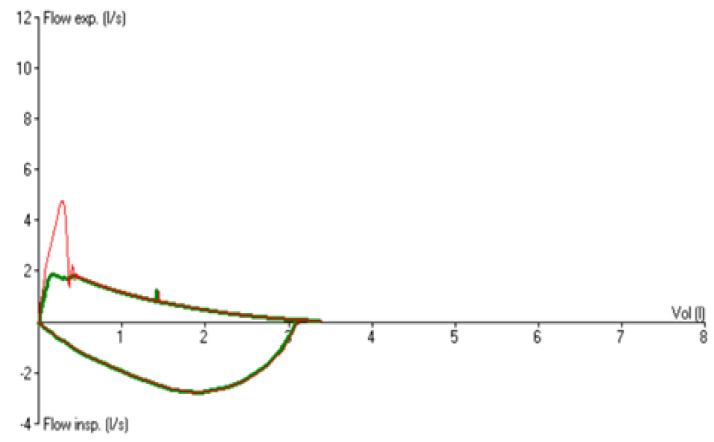
A part of the computer screen during the work of the Tgol.e-spirometry system for e-learning of spirometry. An example that may explain to students the h-shaped flow–volume loop that is observed in severe obstructive lung diseases. The thin line is the loop observed by physicians, and the bold line is the loop that would be if only airflow from lungs could be observed (the difference between both loops is caused by air exhaled from collapsing bronchi at the beginning of the forced expiration).

**Figure A2:**
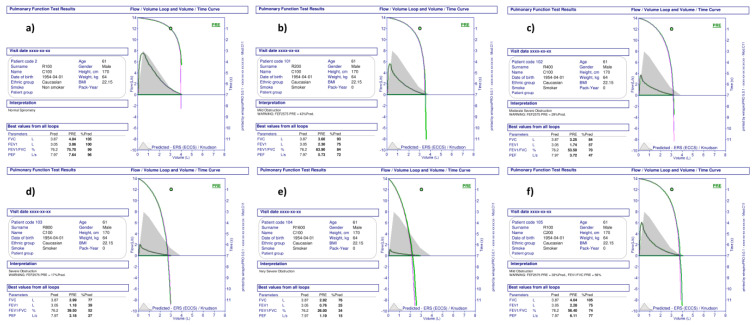
Spirometry measurements of the artificial patient (the hybrid respiratory simulator). (**a**) ‘healthy’ patient; (**b**–**e**)—mild, moderate, severe and very severe *COPD* caused by narrowing of bronchi; (**f**) *COPD* of mild severity caused by a decrease of lung tissue elasticity.

**Figure A3:**
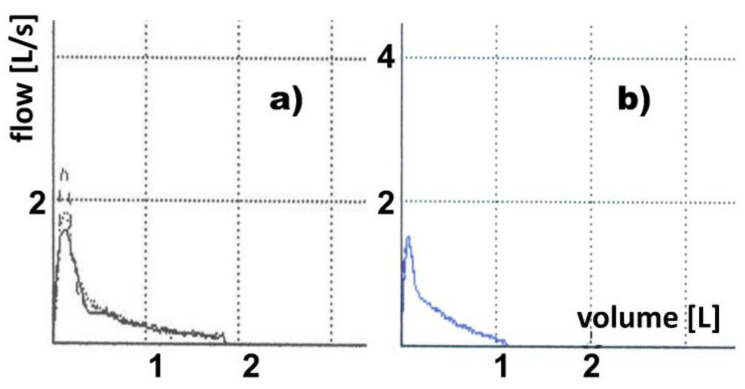
Examples of forced spirometry—scans of spirometer reports. The flow–volume curves registered during three spirometric measurements of real (**a**) and artificial (**b**) patients suffering from severe obstructive lung disease. Spirometry was performed three times in each of these cases. Note the high repeatability in the case of the artificial patient.

## Data Availability

Not applicable.

## Appendix A

Examples of applications of the virtual and artificial patients elaborated by the authors.

### Appendix A.1. Tests of Physical Equipment

An European project entitled “*A remote controlled sensorized artificial heart enabling patient empowerment and new therapy approaches*” (SensorART, no 248763) was a multicenter project focused on the heart failure and innovative telemedicine services. Within this project, the HCVS coupled with other tools developed by other consortium partners was a part of the information and communication system. The control algorithm of the left ventricular rotary CircuLite synergy micro pump (HeartWare, Framingham, MA, USA) was studied by means of this system [62].

In another project, the HCVS was used to investigate the Jarvik 2015 pediatric blood pump, which is a continuous-flow, rotary VAD developed specially for pediatric subjects. It received FDA approval to be used in children with weight 8 kg and more but its application in smaller children was an open issue. Due to the difficulties in testing this pump in small animals corresponding to children <8 kg, the HCVS was used [36]. Several cardiovascular conditions, e.g., the CVS vasodilation or vasoconstriction, left ventricle contractility worsening and recovery, right ventricle failure and contractility improvement as well as bradycardia or tachycardia, for example, were simulated. Simulation results were analyzed in terms of suction and backflow presence, and the capability to maintain adequate patient hemodynamics for different pump speeds and the *CVS* conditions.

The total artificial heart (TAH) is an alternative to heart transplantation due to an increasing number of people succumbing to heart diseases and the lack of heart donors. In this study, the hemodynamic performance of the Realheart^®^ TAH (Scandinavian Real Heart AB, Västerås, Sweden), i.e., a new membrane-based 4-chamber pulsatile cardiac prosthesis device, was evaluated [63]. The TAH was tested under several CVS conditions, including different systemic/pulmonary resistances and compliances as well as for different TAH pumping rates and stroke volumes.

A multilayer membrane failure in pediatric ventricular assist device was investigated [64]. A malfunctioned device connected to the patient was removed and connected to the hybrid cardiovascular simulator. These patient-specific hemodynamic condition was simulated. The device was investigated. The cause of its failure was a rapture of the membrane in the pumping system.

### Appendix A.2. E-Learning

Distal learning may take advantage of models available through the Internet. Therefore, an interactive system for e-learning how to interpret the results of spirometry was developed [23]. E-students could change values of several respiratory system parameters and observe influence of the changes on both the values of forced spirometry indices and the flow–volume loop genesis (Figure A1). Due to the steady development of information technology, a web application containing the whole virtual patient, i.e., including the pulmonary circulation and gas exchange models, was elaborated [16]. In particular, this application enables the end-users to embed their own CVS models. In the frame of the SensorART project mentioned above, a web-based tool was designed for learning purposes. The HCVS was remotely controlled by the system, supporting the learning process of users [24].

### Appendix A.3. E-Support of Medical Decisions and Treatment Optimization (Patient-Specific Approach)

In addition, a specialist decision support system (SDSS) was elaborated in the frame of the SensorART project. The system is a web-based tool offering specialists a set of tools for monitoring, designing the best therapy plan, analyzing data, extracting new knowledge, and making informed decisions. The role of the HCVS is to provide data based on the SDSS requirements (patient-specific hemodynamic data). Two SDSS modules were used to optimize the VAD speed and to detect suction during the LVAD therapy. Web services and file transfer protocols performed the communication between HCVS and SDSS [65].

### Appendix A.4. Cardiopulmonary Interaction (e.g., during Mechanical Ventilation)

The results of simulations performed using the virtual patient surprisingly showed that capillary blood flow was reduced only by low-frequency mechanical ventilation [56]. If the ventilation frequency had a value currently used in a clinical setting, neither the ventilation mode nor minute ventilation would influence blood flow. Unfortunately, the opinion that the pulmonary resistance increases, reducing blood flow during artificial inspiration, is still common (e.g., [66]).

Analysis of model variables showed that separation of the pulmonary circulation from the systemic one by the right and left hearts was crucial. Indeed, the pulmonary resistance depends on vessels cross-section area being proportional to the vessels volume. The total pulmonary blood volume can increase only during the right heart systole and can decrease only during the left heart diastole. If the number of heart cycles during one ventilatory cycle is small, this volume cannot change significantly, and, in consequence, neither the resistance nor the blood flow changes significantly. Only if the ventilator cycle is abnormally long, the pulmonary blood volume may decrease during the inspiration, which causes a pulmonary resistance increase, leading to a blood flow decrease during expiration. More details of this work are presented in [56].

### Appendix A.5. Interpretation of Physiological Phenomena (e.g., Observed during Therapeutic Thoracentesis)

Pleural effusion, i.e., fluid between lungs and the chest wall, is relatively frequently diagnosed, e.g., malignant pleural effusions is present in approximately 15% of patients with cancer [67]. Although thoracentesis is an important diagnostic and therapeutic procedure, there are controversies related to some phenomena observed during and after the procedure. For example, some authors reported PaO_2_ decrease during the procedure, whereas others reported PaO_2_ increase or no significant changes. A latest work showed that all possible PaO_2_ trends can be observed during the procedure, in fact, despite the fact that PaCO_2_ is usually stable. The virtual patient has been used to explain which phenomena and properties of the cardiopulmonary properties are decisive [17]. Simulations showed that the relation in time between the recruitment of collapsed lung parts, pulmonary vessels widening by pleural pressure fall and hypoxic pulmonary vasoconstriction are most important. Additionally, a rare but intriguing and seemingly paradoxical PaO_2_ decrease, not increase, caused by ventilation increase could be explained.

### Appendix A.6. Mechanical Ventilation in Obstructive Lung Diseases

Forced spirometry with the use of the Spirobank G spirometer was performed to verify whether the HRS with the virtual patient as its numerical part could accurately simulate the chronic obstructive pulmonary disease (COPD). The flow–volume curve and the commonly used spirometric indices, i.e., the forced vital capacity (FVC), the forced expiratory volume in 1 s (FEV1), the FEV1/FVC ratio and the peak expiratory flow (PEF), were used to characterize the spirometry results (Figure A2).

Parameters of the virtual patient were matched to the average 60-year-old male of 170 cm in height, which was confirmed by the spirometry results (Figure A2a). Resistive properties of bronchi (i.e., the k value in Equation (2)—see the Section 3.2.1) were increased 2, 4, 8 and 16 times to simulate different COPD severities. Independently, lung tissue compliant properties (i.e., the Compl value in Equation (1)) were increased two times to simulate mild emphysema. The results of spirometry of the HRS and real patients were difficult to be distinguished (e.g., Figure A3). According to the ERS reference values, resistive properties increased 2, 4, 8 and 16 times, which corresponded approximately to the mild, moderate, severe and very severe COPD (Figure A2b–d and e, respectively). The compliant properties increased two times gave results similar to mild COPD.

Then, the artificial patient (i.e., the HRS with the whole virtual patient including models of pulmonary and systemic circulations, and gas transport and exchange) simulated a patient suffering from severe COPD (the resistive and compliant properties were increased 8 and 2 times, respectively) who breathed spontaneously with respiratory muscle effort similar to the healthy subject. The work of breathing was calculated from Campbel’s diagram [68]. The tidal volume was measured by Novametrix CO2SMO + respiratory monitor (Philips), connected to the *HRS* “mouth”. Table A1 shows the values of simulation parameters.

**Table A1:** Breathing parameters and physiological indices during simulation of severe COPD. RR—respiratory rate, TV—tidal volume, IBW—ideal body weight, PEEPi—intrinsic positive end-expiratory pressure, WOB—work of breathing, VD—dead space, FRC—functional residual capacity, RV—residual volume, SpO_2_—arterial oxygen saturation, PaO_2_ and PaCO_2_—arterial oxygen and carbon dioxide tensions, respectively.

RR	TV	TV/IBW	PEEP_i_	WOB	V_D_/TV	FRC	RV	SpO_2_	P_a_O_2_	P_a_CO_2_
[1/min]	[mL]	[mL/kg]	[cmH_2_O]	[J/L]		[L]	[L]	[%]	[mmHg]	[mmHg]
28	190	2.96	4.6	0.68	0.73	4.9	4.0	79	49	63.6

The increased breathing frequency and shallow breaths are typical breathing patterns in COPD [69]. The elevated residual volume and functional residual capacity together with intrinsic PEEP are the results of dynamic hyperinflation caused by expiratory airflow limitation appearing in severe COPD already during tidal breathing [70]. As the lung compliance is significantly nonlinear, the dynamic hyperinflation moves the working point toward a lower lung compliance value. The work of breathing per tidal volume in COPD patients is increased because of the decreased lung compliance and the need for PEEPi overcoming [68]. We observed all those unfavorable phenomena during simulations as well as hypoxia, hypercapnia, a high value of the dead space to the tidal volume ratio, critically low saturation and low tidal volume per the ideal body weight (Table A1). Such values are the respiratory failure indicators [71] and suggest the necessity of ventilatory support.

To analyze this support, a Nellcor Puritan Bennett 840 ventilator (Medtronic, Minneapolis, MN, USA) was connected to the HRS. The three following ventilatory modes were set: the continuous positive airway pressure (CPAP), the biphasic positive airway pressure (BiPAP) and the pressure support ventilation (PSV), which is commonly used in the COPD patients. The CPAP was set at 5 cmH_2_O, the BiPAP was set at 15/5 (high/low pressure) cmH_2_O and the PSV was set at 5 cmH_2_O of extrinsic PEEP (PEEPe) and 15 cmH_2_O of assistance pressure (10 above PEEPe). The results of the simulations were compared with the results of clinical measurements in 22 COPD patients presented by Katz-Papatheophilou et al., who used the same settings of those support modes [71]. The comparison showed good agreement between these results and clinical data (Table A2) despite the fact that the COPD severity in real patients could not be determined with the same precision as in the case of the artificial patient.

**Table A2:** Comparison of simulation results with clinical data from [71]. *BiPAP*, *PSV*, *CPAP*—ventilatory support with the biphasic positive airway pressure, the pressure support ventilation, and the continuous positive airway pressure, respectively. Sim, Lit—results of simulations and clinical, respectively. See the note for the Table A1 for other abbreviations.

	BiPAP		PSV		CPAP	
	Sim	Lit	Sim	Lit	Sim	Lit
WOB [J/L]	1.54	0.46 ÷ 1.6	0.61	0.15 ÷ 1.09	0.7	0.8 ÷ 1.8
PEEP_I_ [cmH_2_O]	6.05	1.14 ÷ 7.14	4.32	0.1 ÷ 6.1	3.31	1.3 ÷ 5.3
TV [L]	0.35	0.21 ÷ 0.59	0.48	0.33 ÷ 0.59	0.3	0.25 ÷ 0.49
T_i_/_Ttot_	0.42	0.36 ÷ 0.44	0.35	0.31 ÷ 0.41	0.4	0.34 ÷ 0.44
P_a_O_2_ [mmHg]	119	80 ÷ 132	125	75 ÷ 121	97	81 ÷ 131
P_a_CO_2_ [mmHg]	38	33 ÷ 61	35	32 ÷ 55	58	36 ÷ 59
